# *In-situ* observation for growth of hierarchical metal-organic frameworks and their self-sequestering mechanism for gas storage

**DOI:** 10.1038/srep12045

**Published:** 2015-07-09

**Authors:** Jung Hyo Park, Kyung Min Choi, Hyung Joon Jeon, Yoon Jung Choi, Jeung Ku Kang

**Affiliations:** 1 Department of Materials Science & Engineering, Korea Advanced Institute of Science and Technology (KAIST), 291 Daehak-ro, Yuseong-gu, Daejeon 305-701, Republic of Korea; 2 Graduate School of EEWS, Korea Advanced Institute of Science and Technology (KAIST), 291 Daehak-ro, Yuseong-gu, Daejeon 305-701, Republic of Korea

## Abstract

Although structures with the single functional constructions and micropores were demonstrated to capture many different molecules such as carbon dioxide, methane, and hydrogen with high capacities at low temperatures, their feeble interactions still limit practical applications at room temperature. Herein, we report *in-situ* growth observation of hierarchical pores in pomegranate metal-organic frameworks (pmg-MOFs) and their self-sequestering storage mechanism, not observed for pristine MOFs. Direct observation of hierarchical pores inside the pmg-MOF was evident by *in-situ* growth X-ray measurements while self-sequestering storage mechanism was revealed by *in-situ* gas sorption X-ray analysis and molecular dynamics simulations. The results show that meso/macropores are created at the early stage of crystal growth and then enclosed by micropore crystalline shells, where hierarchical pores are networking under self-sequestering mechanism to give enhanced gas storage. This pmg-MOF gives higher CO_2_ (39%) and CH_4_ (14%) storage capacity than pristine MOF at room temperature, in addition to fast kinetics with robust capacity retention during gas sorption cycles, thus giving the clue to control dynamic behaviors of gas adsorption.

One of the crucial solutions to energy and environmental problems facing with our humanity could be obtained by discovering a new material or mechanism allowing high capacities with fast kinetics at room temperature for important gas molecules, which include the global warming gases such as carbon dioxide (CO_2_), the cheap and abundant carbon fuels such as methane (CH_4_), and the high energy-carriers such as hydrogen (H_2_). Crystalline porous structures with one specific repeating constructions and high surface areas have been successfully demonstrated as the materials for gas storage at low temperatures^1^,^2^,^3^,^4^, but strategies for making crystals with multiple different constructions as the constituents of their underlying structure, which might be used as gas storage at room temperature, are largely absent.

Most of the approaches to give enhanced capacities for gas storage at room temperature have been focused on increasing affinity of the host structures towards guest molecules by modification of their constructions^5^,^6^,^7^,^8^,^9^,^10^. For example, metal-organic frameworks (MOFs) could facilitate the control of functional groups, pore sizes and open metal sites to adjust the interactions of guest absorbents with the back-bone structures^11^,^12^,^13^. These modifications are still too feeble to give high capacities at room temperature while they function best at low temperatures. On the contrary, for a host structure making strong chemical bonds to capture guest molecules, releasing guest molecules for practical applications requires a very high temperature and it suffers from slow kinetics attributed to its having high activation energy for breaking their strong bonds^14^,^15^. Consequently, a new finding of the structure or mechanism that enables both having high capacities and also releasing them with fast kinetics at room temperature would give a great breakthrough in the field for gas storage. Also, one needs to pursue the goal for realizing gas storage without synthesis of the complex structure having the extremely high surface area and the functional group leading to the undesirable cost.

Here, we report a new finding that enables high capacities for gas storage using a pomegranate MOF (pmg-MOF) having hierarchical meso/macropores only in the core part and enclosed with the microporous crystalline body, in addition to their robust capacity retention with no hysteresis for reversible gas adsorption cycles, where the target molecules occupy first the micropores surrounding meso/macropores and then are captured into these large pore spaces (Fig. 1) with fast kinetics. This so called “self-sequestering storage” mechanism was explored through combination of *in-situ* gas sorption X-ray measurements with molecular dynamics (MD) simulations. In addition, we have elucidated the *in-situ* crystal growth mechanism of the pmg-MOF-5 with the X-ray measurements.

## Results

The pmg-MOF-5 has the unique crystal morphology where a thick crystalline microporous exterior encloses a crystalline meso- and macroporous interior^16^. Hierarchical MOFs usually have different sizes of pores throughout whole crystal structures^17^,^18^,^19^, but pmg-MOF-5 has them in the separated region and has a hierarchical meso/macroporous core enclosed by a microporous shell.

### Formation mechanism of hierarchical pores

**Figure 2:**
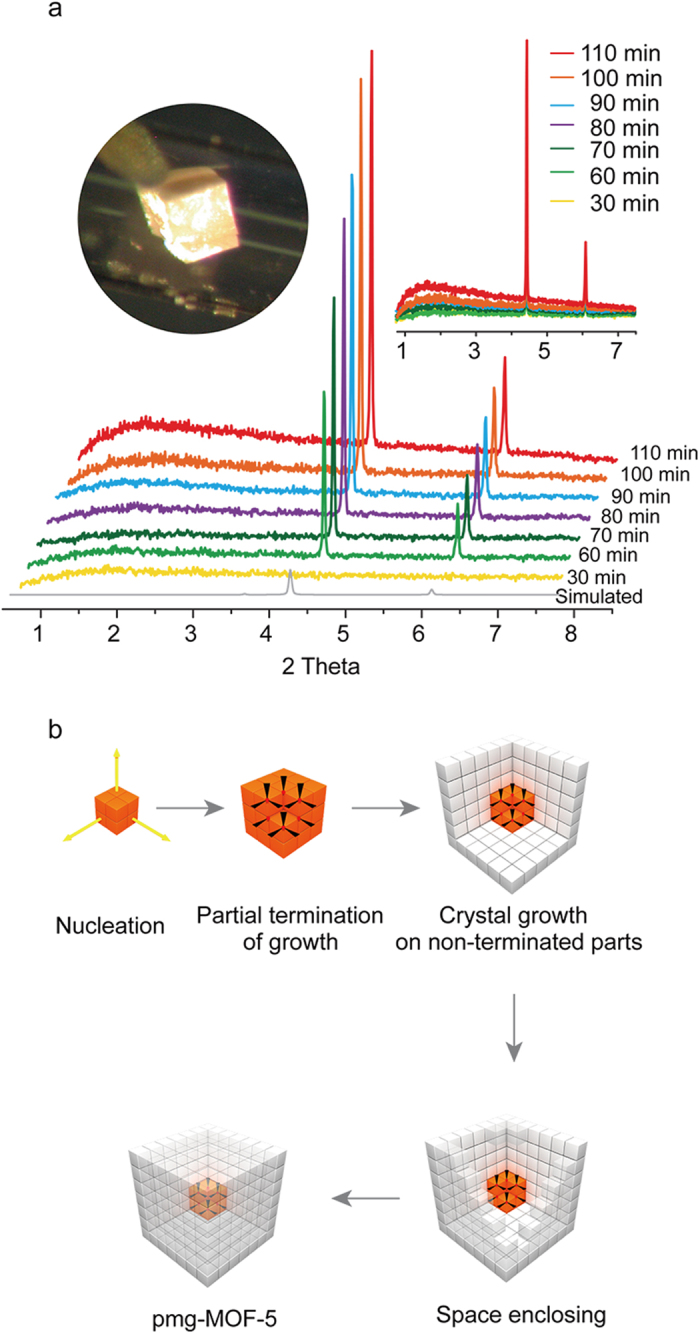
The revelation of growth mechanism for the pmg-MOF-5 using the *in-situ* X-ray measurement. **a,** X-ray diffraction patterns during the time dependent crystal growth. **b,** The illustration for elucidating the growth mechanism of the pmg-MOF-5, where it contains meso/macropores enclosed by micropores.

The formation mechanism of meso/macropores in a crystalline MOF body has been explored using its crystal growth with the *in-situ* X-ray measurements. A stock solution of the MOF was prepared by first dissolving terephthalic acid and zinc nitrate tetrahydrate in *N*,*N*-diethylformamide (DEF), then adding 4-(Dodecyloxy)benzoic acid (DBA) followed by stirring for a day. About 0.1 ml of the stock solution was transferred to borosilica capillary (1 mm diameter) and flame sealed before setting to the X-ray beam line (BL02B2 SPring-8, Japan). The temperature controller was focused on the local capillary part across the beam line and increased to the 150 °C, which gives the pmg-MOF-5 in a desired location (left inset of Fig. 2a) and provides X-ray signals for its nucleation and growth processes. The X-ray data were measured in every 10 minutes interval and shown in Fig. 2a. The first diffraction peaks (2θ = 4.5° and 6.2°) from the crystalline MOF-5 structure were observed after 60 minutes of heating. However, no X-ray scattering (from 1° to 3° in 2θ) was shown in the low angle region. This supports that DBA was not involved yet in the initial nucleation step. The low angle scattering that represents the existence of meso/macropores was getting stronger as the diffraction intensity for the micropore structure was increased from 80 to 100 minutes. When the growth of the pmg-MOF-5 was almost completed at 110 minutes, the low angle scattering of meso/macropores was maximized. These results show that DBA starts to work after nucleation and helps to produce meso/macropore spaces during the crystal growth. Moreover, the changes in the *in-situ* X-ray scattering intensities during the synthesis of the materials, which corresponds to the results in Fig. 2, are quantified in Table S1 of the Supplementary Information. With completion of the pmg-MOF-5 growth, the meso/macropore spaces are enclosed by the outer layer made of the microporous crystalline body, as illustrated in Fig. 2b. The pmg-MOF-5 crystals are washed with organic solvents and dried for activation and then their activated samples along with pristine MOF-5 and DBA were measured by the solid state NMR. The results demonstrate that there are no surfactants remained in the pmg-MOF-5 sample (Figure S2).

### Experimental study of gas sorption mechanism

**Figure 3:**
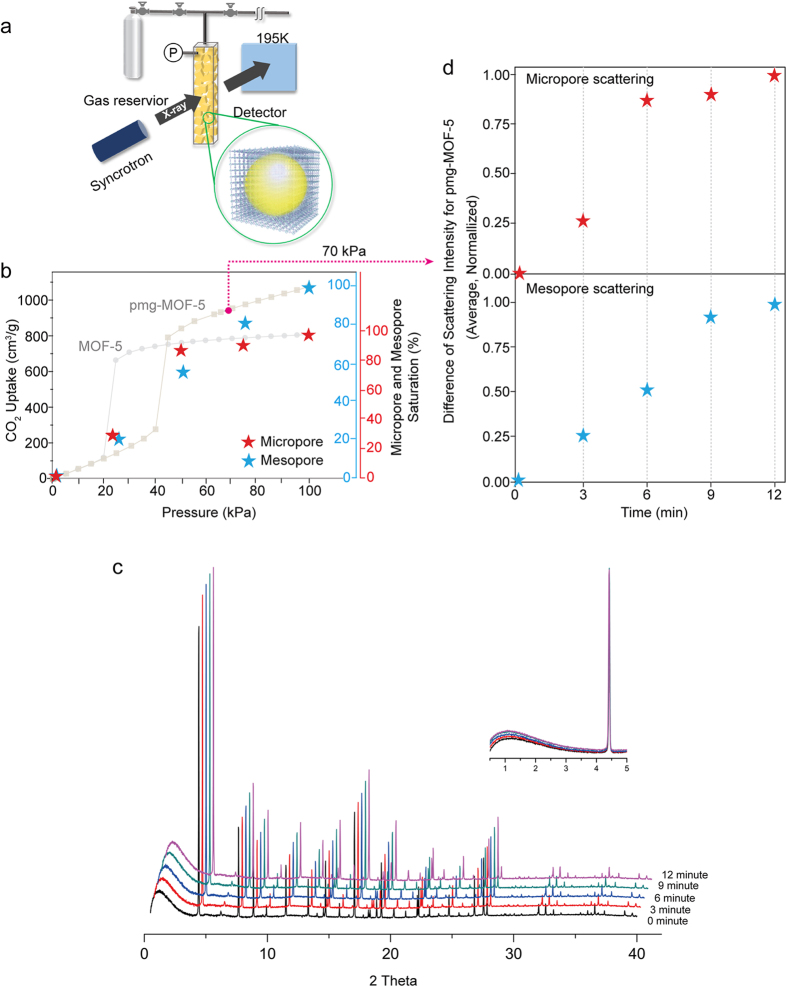
The gas storage mechanism of the pmg-MOF-5 using the *in-situ* X-ray measurement. **a,** The schematic illustration for the *in-situ* gas sorption X-ray measurements using synchrotron (SPring-8, Japan). **b,** The gray graph shows the CO_2_ adsorption of the pristine MOF-5 and the pmg-MOF-5 at a low pressure range of 195 K. The blue stars show the meso/macropore saturation percentages while the red stars show the micropore saturation percentages. **c,** The raw data obtained by the kinetic *in-situ* XRD measurements in different times at 70 kPa. **d,** The scattering intensity of micropores (upper) and meso/macropores (lower) in different times at 70 kPa. At first, the micropores are filled and then the mesopores are filled with CO_2_ as the adsorption proceeds.

In addition, the gas storage mechanism in the pmg-MOF-5 was investigated using the *in-situ* CO_2_ sorption X-ray measurements (BL02B2 beam line, SPring-8, Hyogo, Japan), as shown in Fig. 3a. The comparable electron density of CO_2_ to that of the MOF-5 frameworks and its enhanced CO_2_ sorption at a low temperature make it possible to show a sufficient amount of scattered X-ray signals from micropores and meso/macropores in the pmg-MOF-5^20^,^21^,^22^. The pmg-MOF-5 was prepared in the borosilica capillary (0.4 mm diameter, Charles Supper Company, USA) and aligned in the beam line, while the gas sorption apparatus controls the pressure by dosing different amounts of CO_2_. The capillary cell was mounted to the stainless steel tube using an epoxy attachment. The stainless steel tube was connected to the custom sample holder equipped with the gas dosing and evacuation system. The temperature was accurately controlled by the heat system along with the nitrogen blow. After finishing the mounting of the capillary cell, a leaking test was done by evacuating the capillary cell and dosing the helium gas into the capillary cell. Before the gas adsorption measurement, the pre-treatment was conducted at 150 °C for 1.5 hrs for evacuating other molecules that could exist in the micro-, meso-, and macropores. Also, the X-ray beam with a wavelength 1.0 Å was radiated on the capillary cell for determination of the peak intensity. The adsorption of CO_2_ in meso/macropores could be determined using the integration of the scattered bump in the range from 0.2° to 3.2° in the *2θ* angle, while that in micropores was determined using the peak intensity integrated from corresponding diffraction spots in the IP sheet (Imaging Plate of 400 mm * 200 mm), as shown in Fig. 3.

The pmg-MOF-5 was found to take in a large amount of CO_2_ at a low temperature and its increased X-ray scattering intensities imply that CO_2_ molecules are stored in meso/macropores (Fig. 3b and Table S2 in Supplementary Information). Then, the absorption kinetics in the pmg-MOF-5 was studied by measuring the X-ray scattering at a regular time interval of 2 min with a constant pressure (70 kPa). Figure 3c shows the raw data obtained by the *in-situ* CO_2_ sorption X-ray measurements at different times, which is summarized as the scattering intensity in micro- and mesopore regions (Fig. 3d). This gives a clue about how the different pores work to take in more gas molecules inside meso/macropores (Fig. 3c,d, and Table S3 in Supplementary Information). After dosing CO_2_ molecules to the pmg-MOF-5 sample, the microporous region was saturated during 3 to 6 min (the upper graph in Fig. 3d). After the saturation of micropores, it was found that the scattering in the meso/macropore region was much increased and almost saturated at 9 min. This signals that CO_2_ molecules are saturated at first in micropores and then move into meso/macropores from micropores with fast kinetics. This is possible because CO_2_ molecules diffused into meso/macropores could not pass out through the micropores saturated with gas molecules. This is the self-sequestering gas storage phenomena where gas molecules in large pore spaces have a difficulty in passing dynamically throughout the micropore region and eventually trapped inside.

### Computational study of gas storage mechanism

**Figure 4:**
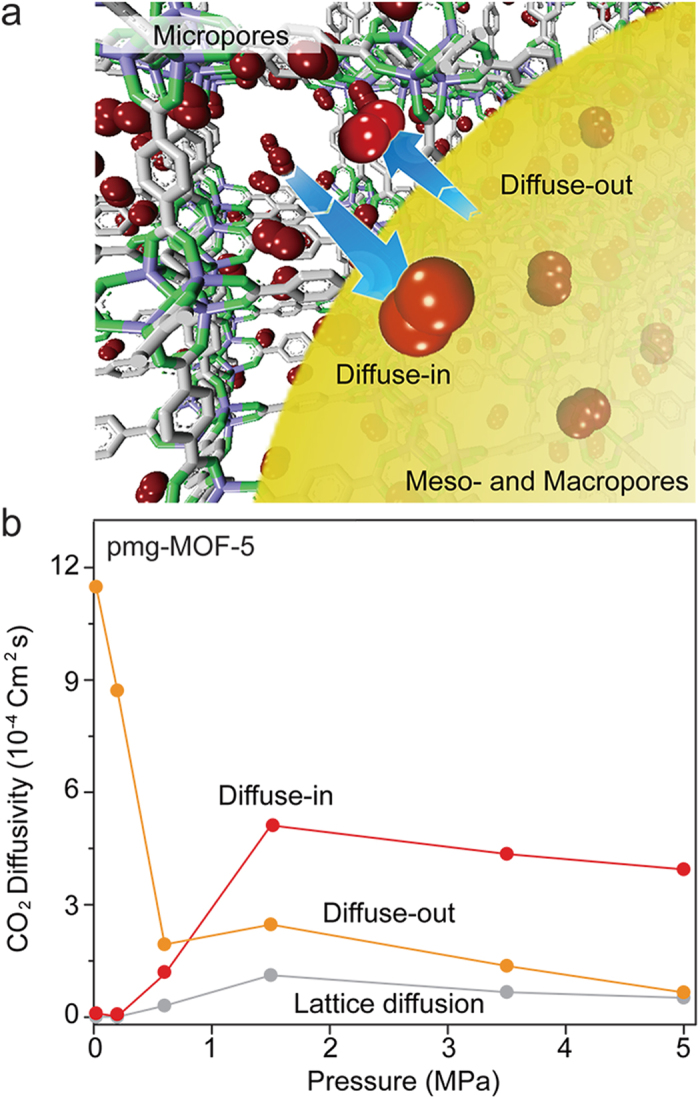
The theoretical values for diffusivities of gas storage in the pmg-MOF-5. **a,** The illustration presenting diffusions at the interface of micropores and meso/macropores, which was determined through theoretical calculations. **b,** Calculation results that show diffusivities for diffuse-in, diffuse-out and lattice diffusion. The diffuse-in is for gas migration from micropores to meso/macropores while diffuse-out is for one from meso/macropores to micropores.

Additionally, we employed the combined simulation of Monte Carlo (MC) and molecular dynamics (MD) simulations to see if similar behaviors and enhancements would be observed at room temperature. After optimizing the number and the position of adsorbed gas molecules in micropores at different pressures using Monte Carlo simulations, the diffusivities of gas molecules are calculated using molecular dynamics simulations in different conditions: (1) lattice diffusion between micropores, (2) diffuse-in from micropores to the meso/macropores, and (3) diffused-out from meso/macropores to micropores, as illustrated in Fig. 4a. The results in a low-pressure region (<1 MPa) show that the diffusivity of diffuse-out is much higher than that of diffusion-in, so that gas molecules are only stored in micropores without using meso/macropores. As the pressure goes up (>1 MPa), the diffusivity of diffuse-in is getting increased and becomes higher than that of diffuse-out, so that gas molecules move into the meso/macropores from micropores and are trapped inside. Meanwhile, we find out that the lattice diffusion remains similar in the whole pressure region. These results support that gas molecules at a low pressure tend to occupy the space in micropores (diffuse-in < diffuse-out). As the pressure is increased, the gas molecules in micropores block the escape of the gas molecules in meso/macropores while they are still available to move into meso/macropores (diffuse-in > diffuse-out). This corresponds to the mechanism observed in the *in-situ* gas sorption X-ray measurements and gives the important clue for enhanced gas storage capacity at room temperature (Fig. 4).

### Gas storage capacities at room temperature

**Figure 5:**
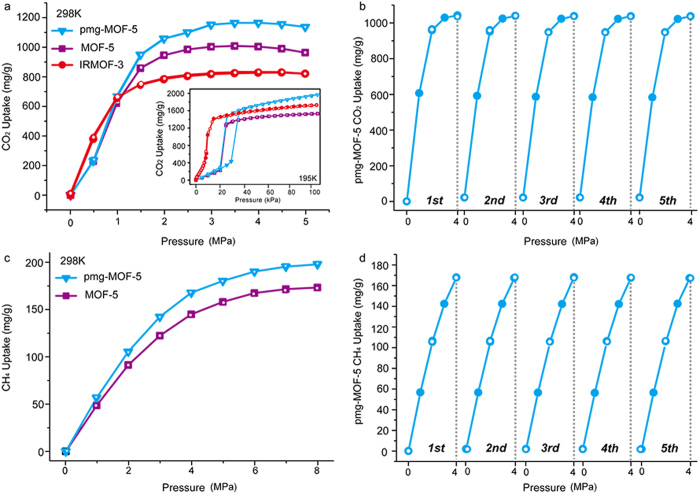
The storage capacities for CO_2_ and CH_4_ adsorption and their corresponding cyclic performance. **a,** The CO_2_ storage capacities of pmg-MOF-5, MOF-5 and IRMOF-5 samples at room and low (inset) temperatures. **b,** The cyclic performance for CO_2_ adsorption cycles in the pmg-MOF-5. **c,** The CH_4_ storage capacities of pmg-MOF-5 and MOF-5 at room temperature. **d,** The cyclic performance of CH_4_ adsorption cycles in the pmg-MOF-5.

We have also explored the capacities of the pmg-MOF-5 for gas storage at room temperature (298 K) using the gravimetric method with the magnetic suspension balance (MSB, Rubotherm), as seen in Fig. 5. It was determined that the capacity of CO_2_ in the pmg-MOF-5 (1136 mg/g) at room temperature was very high compared to the 820 mg/g for the IRMOF-3 (Fig. 5a). This enhanced capacity of about 39% in the pmg-MOF-5 compared to the nitrogen-functionalized MOF-5 (so called as IRMOF-3)^23^,^24^ for CO_2_ uptake at room temperature is very exceptional. It is notable that the IRMOF-3 gives this poor gravimetric CO_2_ sorption uptake capacity at room temperature while it shows the excellent adsorption capacity for CO_2_ molecules at a low temperature (Fig. 5a). Moreover, the sorption behavior of the pmg-MOF-5 at room temperature is found to be similar to that at a low temperature. Also, it shows the similar behavior to that for the pristine MOF-5 in a low-pressure region (<1 MPa) and gives enhancement by using meso/macropores in a high-pressure region (>1 MPa) (Fig. 5a and inset). In addition, we have determined CO_2_ cycling adsorption behaviors and its kinetics at room temperature and these support that the pmg-MOF-5 shows the robust capacity retention with no hysteresis for reversible CO_2_ sorption cycles (Fig. 5b).

CH_4_ is an important guest molecule as an energy-carrier. We have measured the storage capacity for CH_4_ in the pmg-MOF-5 at room temperature to see if it could be also effectively stored. The pmg-MOF-5 (197 mg/g) shows enhanced uptake compared to the pristine MOF-5 (173 mg/g) at 80 bars (Fig. 5c). Considering that the modification in MOF-5 structure does not give much enhancement in the storage capacity of methane^25^, the increased capacity by about 14% compared to that of the pristine MOF-5 is significant. Moreover, the enhancement of CH_4_ uptake in the pmg-MOF-5 is found to be getting more significant in a high-pressure range. These results also support that the self-sequestering mechanism functions well for CH_4_ molecules with negligible atomic dipole moments. Moreover, the pmg-MOF-5 showed robust performance without scarifying kinetics during the reversible CH_4_ sorption cycles at room temperature (Fig. 5d). This implies that the self-sequestering mechanism could provide a new route for realization of high-performance gas storages at room temperature. As the pressure is increased, the pmg-MOF functions to enhance the gas storage capacity by enabling gas molecules to diffuse into the meso/macropores and then by blocking the diffuse-out of molecules from meso/macropores due to the gas molecules occupied in the enclosing micropores.

## Discussion

Consequently, this work represents a simple way of enabling MOFs to have high storage capacity for guest molecules by providing additional gas storage spaces inside their crystals without introducing an extremely high surface area and more delicate chemical modifications of their constituents. Moreover, it gives the clue to control the dynamic behaviors of gas adsorption in micropores and meso/macropores, thus applicable for designing various microporous structures on coupling with meso/macropores for design of high-capacity gas storage materials.

## Methods

### *In-situ* growth X-ray measurement

The mixed solution containing the zinc nitrate tetrahydrate, terephthalic acid and DBA was gently poured into a borosilica capillary (1 mm diameter, Charles Supper Company, USA). The capillary was sealed by a blowtorch and set up in the X-ray beam line SPing-8 (BL02B2, Japan). The temperature controller and the thermocouple were focused on the capillary surface and a crystal growth images were captured by the camera every 10 min.

### *In-situ* gas adsorption X-ray measurement

The XRD patterns with good counting statistics were measured in a synchrotron radiation XRD experiment using an imaging plate as detectors on the BL02B2 beam line at the Super Photon Ring (SPring-8, Hyogo, Japan). The gas pressure control system was used to adjust the pressure of adsorbate. The X-ray wavelength was 1.0 Å.

### CO_2_ and CH_4_ adsorption data

Adsorptions of carbon dioxide (CO_2_) and methane (CH_4_) were measured by a gravimetric method using the MSB (Magnetic Suspension Balances, Rubotherm, Germany), which was equipped with micro-balance and electromagnet for precisely measuring the amount of gas adsorption. See Supplementary Information for details.

### Theoretical calculation

Monte Carlo and molecular dynamics simulations were used to investigate the diffusion behaviors of CO_2_ in MOFs. The whole MC and MD simulations were performed under room temperature, 298 K. See Supplementary Information for details.

## Additional Information

**How to cite this article**: Jung Hyo, P. *et al.* *In-situ* observation for growth of hierarchical metal-organic frameworks and their self-sequestering mechanism for gas storage. *Sci. Rep.* **5**, 12045; doi: 10.1038/srep12045 (2015).

## Supplementary Material

Supplementary Information

## Figures and Tables

**Figure 1:**
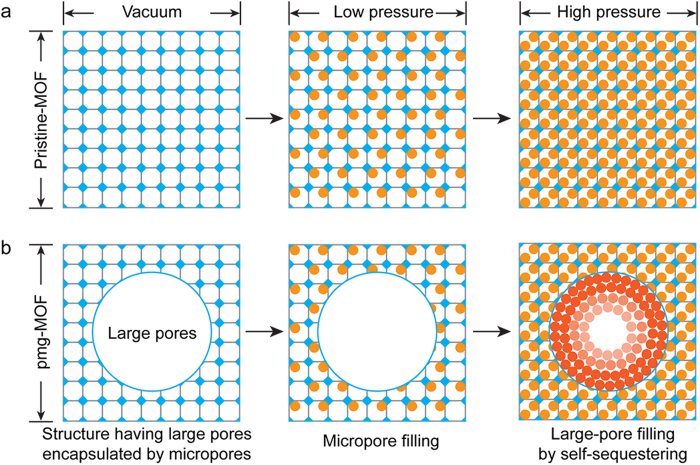
The hierarchy diagram showing the different structures and the gas storage behaviours of the pmg-MOF-5 and the pristine MOF-5. **a**, The pristine MOF-5 takes in gas molecules only in its micropores. b,. The pmg-MOF-5 gives high capacities and fast kinetics using the large pores composed of meso/macropores enclosed by micropores as the pressure of gas molecule is increased, where the filled circles in light ocher and red colors represent schematically gas molecules.
