# Supplementary material for: In-situ observation for growth of hierarchical metal-organic frameworks and their self-sequestering mechanism for gas storage
Source: Sci Rep. 2015 Jul 9;5:12045. doi: 10.1038/srep12045 (PMC4496785; doi:10.1038/srep12045)
Supplement: Supplementary Information [file srep12045-s1.pdf]

Supporting Information

In-situ observation for growth of hierarchical metal-organic frameworks and their self-sequestering mechanism for gas storage

Jung Hyo Park[1, ‡], Kyung Min Choi[1, ‡], Hyung Joon Jeon[1], Yoon Jung Choi[1], Jeung Ku Kang[1,2]*

[1]Department of Materials Science & Engineering and [2]Graduate School of EEWS

Korea Advanced Institute of Science and Technology (KAIST)

291 Daehak-ro, Yuseong-gu, Daejeon 305-701, Republic of Korea

[*]E-mail: jeung@kaist.ac.kr

[‡]These authors contributed equally

Materials and Methods

Materials: All chemicals were purchased from the commercial sources and were used without further purification. The terephthalic acid was obtained from Sigma-Aldrich. $Zn(NO_3)_2 \bullet 4H_2O$ was obtained from Merck. *N,N*-diethylformaide (DEF) and 4-(Dodecyloxy)benzoic acid (DBA) were obtained from Tokyo Chemical Industry.

MOF-5: Zinc nitrate tetrahydrate (392 mg, 1.50 mmol) and terephthalic acid (83 mg, 0.50 mmol) were first dissolved in DEF (5 ml) in a 20 ml vial. A tightly capped vial was placed on the hot plate at 150 °C for 3 hrs to yield the clear crystals. After cooling, the yellow solution was decanted, and the crystals were washed with 20 ml DMF three times. The product was then immersed in chloroform (Merck, 20 ml) for three days, during which time the activation solvent was decanted and freshly replenished three times.

pmg-MOF-5: Zinc nitrate tetrahydrate (333 mg, 1.26 mmol) and terephthalic acid (58.1 mg, 0.350 mmol) were first dissolved in DEF (5 ml) in a 20 ml vial, then DBA (45.9 mg, 0.150 mmol) was added and stirred for a day. A tightly capped vial was placed on the hot plate at 150 °C for 3 hrs to yield dark yellow crystals for the pmg-MOF-5. After cooling, the yellow solution was decanted, and the crystals were washed with 20 ml DMF three times. The product was then immersed in chloroform (20 ml) for three days, during which time the activation solvent was decanted and freshly replenished three times.

IRMOF-3: Zinc nitrate tetrahydrate (392 mg, 1.50 mmol) and 2-aminoterephthalic acid (90 mg, 0.50 mmol) were first dissolved in DEF (5 ml) in a 20 ml vial. A tightly capped vial was placed on the hot

plate at 150 °C for 5 hrs to yield the clear crystals. After cooling, the yellow solution was decanted, and the crystals were washed with 20 ml DMF three times. The product was then immersed in chloroform (Merck, 20 ml) for three days, during which time the activation solvent was decanted and freshly replenished three times.

Characterization

BET measurement using nitrogen at 77K :

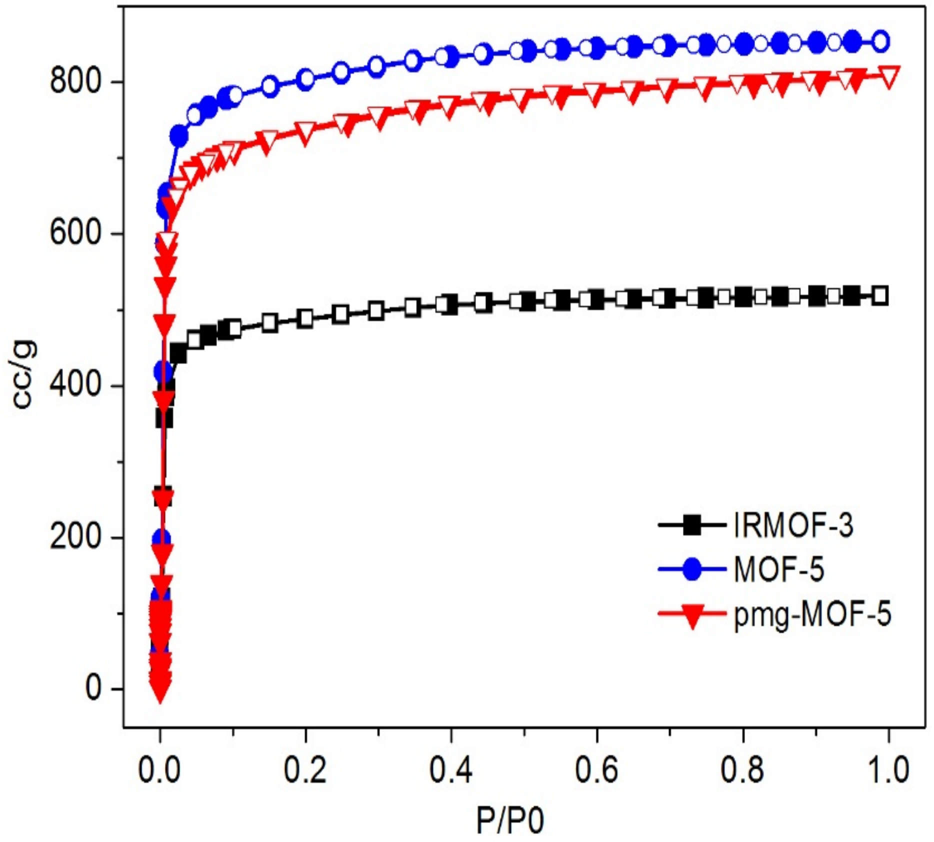

Figure S1 | BET measurement of MOF-5, pmg-MOF-5 and IRMOF-3 using nitrogen gas at 77K.

Solid state NMR of pmg-MOF-5, MOF-5, and DBA.

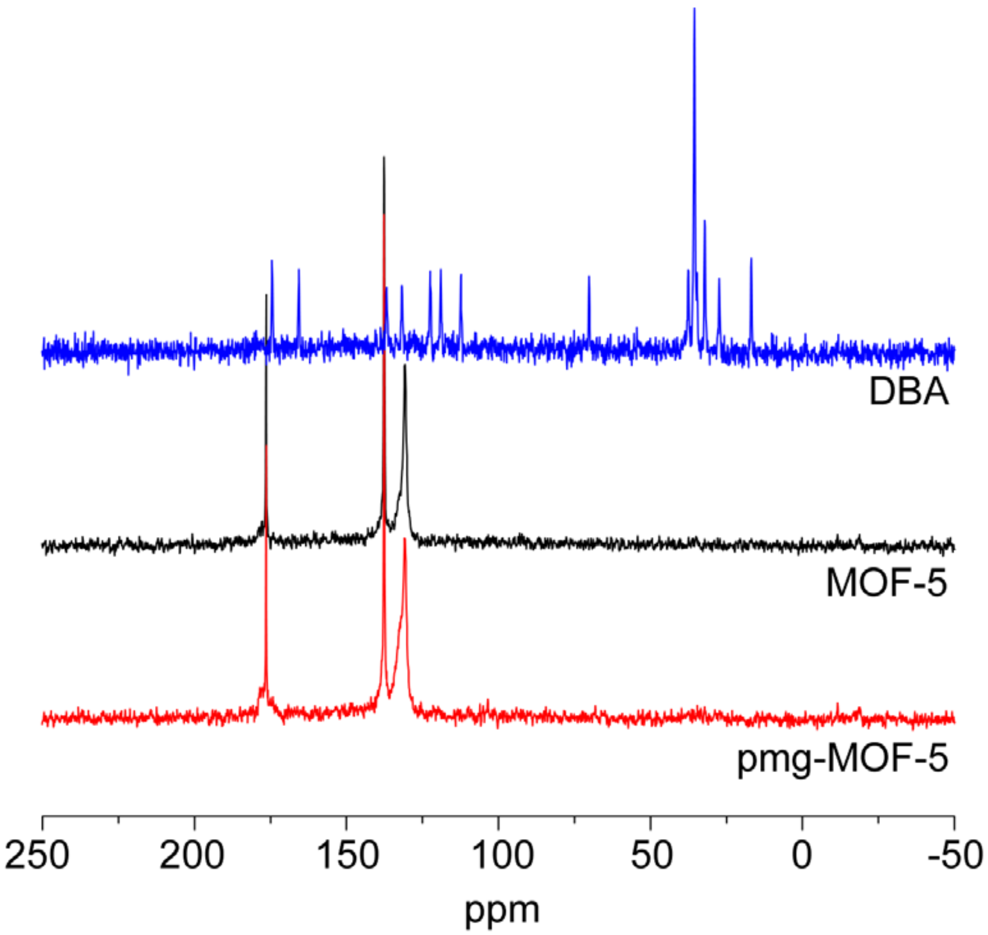

Figure S2 | Solid state NMR results of pmg-MOF-5, MOF-5, and DBA.

Measurement details

Details for *in-situ* X-ray measurements: An *in situ* synchrotron powder diffraction experiment of gas adsorption was carried out at the beamline BL02B2 SPring-8, Japan. In order to perform an *in situ* measurement in the gas adsorption state, a sample holder and a gas handling system were constructed and equipped with the standard experimental settings in the beam line. The sample cell must isolate the powder sample from the ambient atmosphere. The temperature and gas pressure must be controlled to obtain various adsorption states. With a length of approximately 2 mm, the powder sample is enclosed by a thin-walled glass capillary. In a typical case for MOF, the size of the capillary is 0.4 mm with an inner diameter for the wavelength of an incident X-ray of 1 Å. The capillary containing the powder specimen was attached to the stainless steel tube using an epoxy adhesive. The capillary was mounted to the custom sample holder, which was constructed from Swagelok parts and the steel tube, for the gas introduction. A stainless steel tube attached to the sample holder was connected to the gas handling system with a number of valves and a pressure gauge. The temperature of the sample was controlled by a high and low temperature nitrogen gas blower. In our experimental with the pmg-MOF-5, the atmosphere inside the capillary is evacuated, and a helium leak detector is used to confirm that there are no leaks in the system. A vacuum leak causes the interfusion of water molecules, which may cause demolishing of MOF. Before the measurement in gas adsorption, the water molecules or organic solvent must be removed from the nanopores during evacuation (for 1.5 hrs at 150 °C). The CO_2 was then introduced at 70 kPa pressure, 195 K. In order to obtain a kinetic sorption data, XRD was measured at the 2-minute interval. In case of mesopores adsorption, the adsorption of CO_2 was presented as the integration of scattered bump in the range from 0.2° to 3.2° in 2θ after fitting. For the micropore adsorption, direct integration of each diffraction spots in IP sheet at different pressure gave us the accurate comparison for the pmg-MOF-5 (An imaging plate (IP) of 400 mm × 200 mm was used as a detector covering the range from 0° to ~70° in 2θ). The scattered intensity ratio at target pressure is the relative value to the peak intensity at 0 kPa in same specimen. Slight increase in the range of mesopores was occurred by scattering of gas molecules in the measuring sample cell.

Table S1. Quantitative changes in the *in-situ* X-ray scattering intensities during the synthesis of the materials.

	Meso/macropores		Micropores				
Peak position (2θ)	0.2-3.2		4.4		6.3		Ave
Time (min)	I[1]	R[2]	I[1]	R[2]	I[1]	R[2]	R[3]
30	0	**0**	0	**0**	0	**0**	**0**
60	17	**2**	103	**37**	37	**42**	**40**
70	2	**0**	163	**60**	58	**66**	**63**
80	196	**22**	196	**72**	68	**77**	**74**
90	390	**44**	236	**86**	79	**89**	**88**
100	491	**55**	261	**95**	82	**93**	**94**
110	887	**100**	274	**100**	89	**100**	**100**

[1]Intensity integrated from peaks in meso/macropores and micropores. [2]Ratio (%) of the peak intensity change from 30 min to 110 min. [3]Average ratio of the peak intensity change for the micropore region.

Table S2. Quantitative XRD intensities of micropores and meso/macropores from 0 to 100 kPa.

	Meso/macropores		Micropores						
Peak position (2θ)	0.2-3.2		6.3		13.2		17.1		Ave
Pressure (kPa)	I[1]	R[2]	I[1]	R[2]	I[1]	R[2]	I[1]	R[2]	R[3]
0	204	**0**	938	0	211	0	219	0	**0**
25	873	**18**	980	20	229	18	226	12	**49**
50	1817	**44**	1140	95	288	75	261	70	**241**
75	2518	**63**	1144	97	292	79	266	79	**255**
100	3865	**100**	1151	100	314	100	278	100	**300**

[1]Intensity integrated from peaks in meso/macropores and micropores. [2]Ratio (%) of the peak intensity change from 0 to 100 kPa. [3]Average ratio of the peak intensity change for the micropore region.

Table S3. Quantitative XRD intensities of micropores and meso/macropores on different times at 70 kPa.

	Meso/macropores		Micropores						
Peak position (2θ)	0.2-3.2		4.4		8.9		11.5		Ave.
Time (min)	I[1]	R[2]	I[1]	R[2]	I[1]	R[2]	I[1]	R[2]	R[3]
0	2150	**0**	627	0	135	0	119	0	**0**
3	2353	**29**	650	53	136	33	120	17	**34**
6	2542	**56**	670	96	140	120	124	90	**102**
9	2780	**89**	670	97	139	89	124	93	**93**
12	2857	**100**	672	100	139	100	124	100	**100**

[1]Intensity integrated from peaks in meso/macropores and micropores. [2]Ratio (%) of the peak intensity change from 0 to 12 min. [3]Average ratio of the peak intensity change for the micropore region.

Details for high-pressure CO_2 sorption measurements: The high-pressure gases adsorption of carbon dioxide were measured by a gravimetric method, MSB (Magnetic Suspension Balances, Rubotherm, Germany). The MSB was equipped with the micro-balance and the electromagnet to enable a precise measurement of the amount for adsorption. Also, to obtain the adsorption isotherm, the data points are corrected for two independent buoyancies: buoyancy from the empty vessel components of the gravimetric measuring system and buoyancy from the adsorbent material. The buoyancy of the empty vessel components is applied based on the empirically determined volume of vessel components. The empty vessel measurement with inert gas gave the linear plot of weigh and gas density by the change of gas pressure. The slope of that graph that is the quotient of weight change in the empty vessel ($\Delta W_{empty\ vessel}$) and the inert gas density change ($\Delta d_{inert\ gas}$) gave the experimental empty vessel volume ($V_{empty\ vessel}$).

$$V_{empty\ vessel} = \frac{\Delta W_{empty\ vessel}}{\Delta d_{inert\ gas}}$$

The product of this empty vessel volume and the correlated density of the measuring gas ($D_{measuring\ gas}$) determine the weight lost due to the buoyancy of the empty vessel component ($W_{buoy,\ empty\ vessel}$).

$$W_{buoy,\ empty\ vessel} = D_{measuring\ gas} * V_{empty\ vessel}$$

The adsorbent material buoyancy is applied based on the theoretical crystallographic density of the adsorbent framework and the pressure-dependent density of the pure gas. The crystallographic density of the organic frame ($D_{organic\text{-}frame}$) in MOF-5 is determined by the same method in the previous paper [S1]. The crystallographic density of the pore-directing agent frame ($D_{macro\text{-}frame}$) in the pmg-MOF-5 structure was determined from the formulas weight of the crystallographically evacuated model ($FW_{macro\text{-}frame}$) and the frame backbone volume ($V_{bb,\ macro\text{-}frame}$) as calculated in the Void Space routine in Cerius2 using the defaults (medium grid, 1.4 Å probe radius).

$$D_{macro\text{-}frame} = \frac{FW_{macro\text{-}frame}}{V_{bb,\ macro\text{-}frame}}$$

The quotient of the initial evacuated mass for the adsorbent to its crystallographic density gives the

framework volume of the experimental sample. In the case of the pmg-MOF-5, the total framework volume (V_{total}) is the sum of the volume of organic linker frame ($V_{organic\text{-}frame}$) and the pore-directing agent frame ($V_{macro\text{-}frame}$), as expressed by the following equation of

$$V_{total} = V_{organic\text{-}frame} + V_{macro\text{-}frame}$$
$$= \frac{W_{pmg\text{-}MOF\text{-}5}}{D_{organic\text{-}frame}} * P_{organic\ linker} + \frac{W_{pmg\text{-}MOF\text{-}5}}{D_{macro\text{-}frame}} * P_{macropore\text{-}directing\ agent}$$

where $P_{macropore\text{-}directing\ agent}$ is the portion of pore-directing agent in the pmg-MOF-5 and $P_{organic\ linker}$ is the portion of organic linker in the pmg-MOF-5. The product of this framework volume and the correlated density of the gas (D_{gas}) gives the weight of the gas displaced, which is the weight lost due to the buoyancy of the adsorbent ($W_{buoy,pmg\text{-}MOF\text{-}5}$) using the following equation of

$$W_{buoy,pmg\text{-}MOF\text{-}5} = V_{total} * D_{gas}$$

The weight losses due to the buoyancy of the empty vessel components ($W_{buoy,\ empty\ vessel}$) and of the sample ($W_{buoy,sample}$) are added back in to the experimental weight to obtain the adsorption data point on the isotherm.

Details for computational calculations: In this work, Monte Carlo (MC) and Molecular Dynamics (MD) simulations were used to investigate the diffusion behaviors of CO_2 in MOFs. The whole MC and MD simulations were performed under room temperature, 298K. The carbon dioxide molecules was modeled to the linear and fixed bond length C–O of 1.161 Å from the experimental value. The point charges 0.6645 e and –0.33225 e taken from the experimental values [S2] was also placed on the carbon and oxygen atoms, respectively, to approximate the quadrupole moment of CO_2. All the MOFs studied in this work were treated as rigid with atoms frozen at their crystallographic positions during simulations. The simulation cell consists of the 1x1x1 elementary cell of the MOFs and the structural model for a unit cell of MOFs was constructed using the experimental XRD data (a, b, c= 25.669 Å, α, β, γ=90.0º)[S3]. The crystalline structure of the pmg-MOFs was derived from the ideal structure of MOFs, whose unit cell is composed of MOFs having a and b lattice sizes of 25.669 Å and a vacuum

slab of 30.0 Å along the c-direction. The dangling bonds in the pmg-MOFs were terminated with hydrogen atoms. Each atom in CO_2, and MOFs was represented as a Lennard-Jones (LJ) interaction site, for which the LJ potential parameters were taken from the Universal force field (UFF)[S4]. The periodic boundary conditions were applied in all three dimensions. In both MC and MD simulations, a cutoff distance of 12.8 Å was used for the LJ interactions. All the MC and MD simulations were performed by the Sorption (MC) module and Forcite (MD) module in the Material Studio 4.1 software suite [S5].

Before the MD computations, MC simulations were performed using the fixed loading method, in order to generate 10 initial configurations with randomly distributed CO_2 molecules. MC simulation consists of $1x10^6$ equilibrium steps followed by $2x10^6$ production steps. The value of a fixed loading (i.e. a number of adsorbed molecules) at a given pressure and 298K were determined from the experimental adsorption isotherms. In each fixed loading calculation, we chose ten configurations which are at the lowest energy states.

The finally obtained configuration was then used as initial configuration of a MD run under 298K and overall density. The MD simulations were performed in the canonical ensemble (NVT) with the time-step of 1 fs. The initial velocities were randomly generated according to a Boltzmann distribution at a given temperature and the the Berendsen thermostat was used to maintain the constant-temperature condition[S6]. MD simulation consists of $5x10^5$ steps to equilibrate the system followed by $2x10^6$ steps to sample the diffusion properties of interest. The coordinates of a configuration during final $2x10^6$ MD steps were stored every 1.0 ps (every 100 time steps).

The self-diffusion coefficient D_α is defined using the Einstein expression[S7] of

$$D_\alpha = \frac{1}{6N_\alpha} \lim_{t\to\infty} \frac{d}{dt} \sum_{i=1}^{N_\alpha} \langle [r_i(t) - r_i(0)]^2 \rangle$$

where r_i denotes the position vector of atom α, and the angular brackets denote averaging over all choices of time origin within a dynamics trajectory. The limiting slope of the mean square

displacement (MSD) as a function of time can be used to evaluate the self diffusion coefficient of an atom undergoing a random Brownian motion in three dimensions [S8]. From the stored configurations of each trajectory, the MSD curves were calculated.

Supporting References:

[S1] Millward A. R., Yaghi O. M. Metal−Organic Frameworks with Exceptionally High Capacity for Storage of Carbon Dioxide at Room Temperature. *J. Am. Chem. Soc.* **127**, 17998-17999 (2005).

[S2] Babarao S., Jiang J. Molecular Screening of Metal−Organic Frameworks for CO_2 Storage. *Langmuir* **24**, 6270-6278 (2008).

[S3] Hailian L., Eddaoudi M., O'Keeffe M., Yaghi O. M. Design and synthesis of an exceptionally stable and highly porous metal-organic framework. *Nature* **402**, 276-279 (1999).

[S4] Rappé A. K., Casewit C. J., Colwell K. S., Goddard III W. A., Skiff W. M. UFF, a full periodic table force field for molecular mechanics and molecular dynamics simulations. *J. Am. Chem. Soc.* **114**, 10024-10035 (1992).

[S5] Material Studio 4.1, Accelrys Software Inc.

[S6] Berendsen H. J. C., Postma J. P. M., van Gunsteren W. F., DiNola A., Haak J. R. Molecular dynamics with coupling to an external bath. *J. Chem. Phys*. **81**, 3684 (1984).

[S7] Kärger J., Ruthven D., Kärger J., Ruthven D. Diffusion in Zeolites and other Microporous Materials, *Wiley*, New York (1992).

[S8] Frenkel D., Smit B. Understanding Molecular Simulation: From Algorithms to Applications, *Academic Press*, San Diego (2002).
